# Linking the preference in a bilateral asymmetric task with handedness, footedness, and eyedness: The case of ice-hockey

**DOI:** 10.1371/journal.pone.0294125

**Published:** 2024-05-23

**Authors:** Simon Grondin, Daniel Fortin-Guichard, Charles-Anthony Dubeau, Émie Tétreault

**Affiliations:** École de Psychologie, Université Laval, Québec, Québec, Canada; The Ohio State University, UNITED STATES

## Abstract

Most people know whether they are left-handed or right-handed, and usually base this assessment on preferences during one-handed tasks. There are several manual tasks that require the contribution of both hands, in which, in most cases, each hand plays a different role. In this specific case, holding an ice-hockey stick is particularly interesting because the hand placement may have an incidence on the playing style. In this study (*n* = 854), the main objective was to determine to what extent the way of holding an ice-hockey stick is associated with other lateralized preferences. Amongst the 131 participants reporting a preference for the left hand in unilateral tasks, 70.2% reported a preference for shooting right (placing the right hand in the middle of the stick); and amongst the 583 participants reporting a preference for writing with the right hand, 66.2% reported a preference for shooting left. 140 (16.4%) participants were classified as ambidextrous and 61.4% of them reported a preference for shooting right. This preference on the ice-hockey stick is closely correlated (uncrossed preference) to the way one holds a rake, shovel, or broom, or a golf club, but inversely related to the way one holds an ax and a baseball bat. The link between the way of holding the ice-hockey stick and eyedness or footedness is weak. These results are contrasted with the results reported by Loffing et al. (2014) and reveal the need to clarify the exact nature and requirements of the targeted tasks when studying bilateral asymmetric preferences.

## Introduction

Most people exhibit a preference for using one hand over the other when executing a manual task such as holding a spoon while eating. Some researchers report that approximately 10% of people prefer using their left hand [1], and others report that this proportion varies between 10 and 13% [2]. Indeed, depending on the criteria adopted to define hand preference, left-handedness prevalence varies between 9.3% and 18.1%, with 10.6% being a good overall estimate [3]. Also, approximately 10% of people show no hand preference (i.e., ambidextrous) [4, 5], although they were typically neglected in scientific studies by being included with left-handers [6, 7]. Moreover, there is a documented sex difference for handedness, with males being more often ambidextrous or left-handers than females [8].

Lateral preference is not limited to activities involving the contribution of hands. There is notably preference for using one foot over the other, or one eye over the other. As for footedness, meta-analyses revealed that 12.1 to 23.7% (large estimates corresponding to cases where left- and mixed-footers are taken as a single, non-right group) are left-footed [9]. As was the case for handedness, males are more often left-footed than females [9]. Also, 60.1% of left-handers are left-footed, and this percentage decreases to 3.2% for right-handers [9]. The proportion of people that have a preferred eye is not as systematically lateralized as it is for handedness or footedness. Overall, approximately 28.6% of people show a left-eye preference. More specifically, 24.2% of people who throw and write with their right have a left-eye dominance, and 72.3% of people who throw and write with their left hand have a left-eye dominance [10].

Lateral preferences can also be observed in a category of behavior where both limbs, or both hands, contribute to the action or task (note that, even in a motor task such as throwing, the non-preferred limb contributes to the mechanical efficiency of the motion). Hereby, we will refer to uni- vs. bimanual tasks to distinguish between tasks that require one hand, in the former case, and tasks that require both hands, in the latter case. In bimanual activities, the role of hands could be symmetrical, meaning both hands have the same role, as when climbing a ladder (or when swimming or cycling, to take sport-related examples), or asymmetrical, meaning the hands do not have the same role, like when distributing playing cards (or when swinging a golf club to give a sport-related example) [11]. According to Guiard, in bimanual tasks where both hands are assigned different roles, there are three principles of cooperation between the hands [11]. In other words, instead of simply describing one hand as more skilled, or more involved in an asymmetrical bilateral task, Guiard proposes a logic of partition of labor (a kinematic chain approach) between the hands. The principles stipulate that 1) the dominant hand (the one most often observed in unimanual tasks and revealing a lateral preference) is more likely to be assigned to a portion of the task requiring fine resolution (spatial or temporal), with the nondominant hand playing a supporting role; 2) a frame of reference provided by the non-preferred hand guides the motion executed by the preferred hand (i.e., the nonpreferred hand delineates a spatial frame into which the activity of the preferred hand inserts contents. For instance, a right-handed person will use the left hand to hold the needle and the right hand to pass the thread); and 3) the non-preferred hand acts first, and the preferred hand acts subsequently.

### Predicting lateral preferences

There is a substantial body of literature on the links between handedness preferences in a variety of unimanual tasks. Overall, the preferred hand in one unilateral task is strongly related to the preferred hand in another unimanual task. For instance, following their collection of data from several studies, McManus et al. reported that only 1.6% of people who write with the right hand prefer throwing left, while 28.8% of people who write with their left prefer throwing right [10]. These data are relatively consistent with the ones reported in a large survey where 1.55% of male and 1.05% of female right-handed writers prefer throwing left, and 32.4% of male and 37.5% of female left-handed writers prefer throwing right [6]. This shows that cross-lateral preference for unimanual tasks is more frequent in left-handed writers than it is in right-handed writers.

The links between lateral preferences, as established by the Edinburgh Handedness Inventory (EHI) [12], and sport-specific unimanual tasks are also very high [13]. For instance, for fencing and racket activities, about 85% of left-handers will use their left hand, and 99% of right-handers will use their right hand. Data from the same study indicate that for bimanual asymmetrical sport activities, the picture is not as clear. About 90% of right-handers will bat right in baseball, and about 66% of left-handers will bat left. For golf, about 82% of right-handers will play right, and about 61% of left-handers will play left.

These inconsistencies between preference in unilateral tasks and bilateral tasks like golf and baseball indicate that asymmetrical sport activities offer a different viewpoint on the study of laterality. In the case of golf, there are reasons to believe that the placement of hands on the club will impact performance. While most people opt for a congruent approach (e.g., play right for right-handers), better players are much more likely to adopt a cross-lateral (or “reversed stance”) approach (playing left for right handers for example) [14]. In cricket, a somewhat similar result was reported. While 9% of inexperienced male cricketers adopted a cross-lateral preference, 40% of the 43 professionals tested did [15]. In baseball, the way of placing hands also has an impact on performance [16–18]. It has been shown that amongst Major League players batting left, left-handers are more likely than right-handers to hit with power, but also to strike out [17]. In terms of Guiard’s model, left-handers batting left use the right, non-preferred, hand to provide the reference; this non-preferred hand acts first and is involved in the micrometric component of the swing. Guiard had hypothesized that swinging left when being left-handed would optimize power. This was later demonstrated in baseball and ice-hockey [17, 19].

### The case of ice-hockey

Ice-hockey is another asymmetrical bilateral sport activity. It is of particular interest from a laterality viewpoint since the organization of the game itself presents a lateral component (having left or right wingers, left or right defenders). In addition, the game is dynamic and requires a continuous adaptation of the position occupied on the ice rink [20]. Moreover, contrary to activities such as batting in baseball or swinging with a golf club, the position of hands on the ice-hockey stick are not close to each other, as they do not touch each other (ice-hockey should not be confused with field hockey where the stick is much shorter and each player, by rule, shoots from the right). This way of holding the stick results in different constraints or possibilities. In ice-hockey, players have to accommodate the need for shooting with power, as well as the need for control and finesse in stick handling.

There is not much data about the shooting side preference of left- and right-handed ice-hockey players. Traditionally, in ice-hockey, more players shoot left (left hand near the middle of the stick, and right hand at the extremity of the handle, i.e., with the stick at the left of the shooter) than right (right hand near the middle of the stick and left hand at the extremity of the handle, i.e., with the stick at the right of the shooter). For example, the percentage of left-shooters in the *National Hockey League* (NHL) increased from 57% between the years 1917–1926 to 67% between 1997 and 2006, and varies slightly depending on position, forwards versus defensemen [21]. Assuming that most people are right-handers, this means that a majority of NHL ice-hockey players likely adopt a cross-lateral preference. That is not what Guiard’s model would predict [11]. Placing the nonpreferred hand at the extremity is a stance generally adopted when using a two-handed hitting implement [22]. In other words, most people opt for using their nonpreferred hand at the extremity to provide the reference, to act first, and be involved in the macrometric component of the task. That is the case of sports like batting in baseball and driving in golf where swinging is an important component of the task (the efficiency when swinging depends on oculo-manual coordination in baseball, and on control when driving. But in both cases, there are generally benefits to hit the ball with force and rapidly [i.e., to exert power] because the ball will travel a longer distance). Apparently, the question of hand placement in ice hockey cannot be posited as simply, at least when we look at the NHL data. In addition to having to hit the puck with power, there is a need in ice hockey for keeping and controlling the puck (stick handling) and moving with it.

Only a few studies directly tested the links between the lateral preference for unimanual tasks and the preference when holding an ice-hockey stick. In a study involving 194 male ice-hockey players, 55.6% of those with a left-hand preference (*n* = 27) were shooting right, and 67.1% of those with a right-hand preference (*n* = 167) were shooting left [19]. In this study, handedness was assessed with the EHI. In a survey of 214 men that were mainly playing golf or baseball, 65.7% of left-handers were shooting right and 55.9% of 179 right-handers were shooting left [23]; the handedness in this study was based on responses to five unimanual tasks, namely write, draw, brush teeth, throw a ball, and cut with scissors [24]. These samples were however small, resulting in a very small number of left-handed participants.

In a more recent study of a German sample of 812 participants, Loffing and colleagues [13] rather found that the correlation (point biserial) between the preferred hand in a unilateral task (based on the German version of the EHI) and the position of hands on the ice-hockey stick was positive (.254), and this link was stronger for women (.328) than for men (.175). In their study [13], out of 327 male right-handers, 249 (76.15%) shot right, and out of 409 female right-handers, 313 (76.53%) shot right. Amongst the left-handers in Loffing et al. [13], 50% out of 38 males and 73,68% out of 38 females shot left. These data contradict what was reported in the previous literature [19, 23], a fact that might be explained by the non-familiarity of German participants with the requirements in ice-hockey. However, this explanation has limitations considering that participants in this study were presented with images of a person performing the required motor tasks (see the photo related to ice-hockey in their Table S1).

### The present study

There are inconsistencies in the literature concerning the relationship between handedness and the way of placing hands on a stick for playing ice-hockey. The first objective of the present study is to determine to what extent unimanual activities are related to the placement of hands on an ice-hockey stick. Because most participants are Canadian, more cross-lateral cases are expected than uncrossed cases. The second objective of the study, admittedly exploratory, is to examine the relationship between gripping an ice-hockey stick and two other types of lateral preference not related to handedness, namely footedness and eyedness. Since the dominant eye does not explain the success of cross-lateral preference in other sports such as cricket and golf [14, 15], we do not expect to find high correlations between the preferred eye and the stance adopted in ice-hockey.

The investigation of the relationship between lateral preference in ice-hockey and other activities is extended to other asymmetrical bilateral sport and non-sport activities. In a previous study on the hand placement in ice-hockey, it was shown that players with a cross-lateral preference (righthanders shooting left or lefthanders shooting right) were more likely to emphasize a playing style based on control, and players with a same-lateral preference (lefthanders shooting left or righthanders shooting right) were more likely to emphasize a playing style based on power [19]. Therefore, cross-lateral preference should be negatively linked with hand preference of bimanual activities like golf or baseball requiring power, and positively linked with hand preference of bimanual activities like raking not requiring power.

There are two more important objectives in the present study. One is to document the case of left-handers. The size of the samples of left-handers is generally small in studies on hand placement in asymmetrical bimanual activities. There are also less consistencies in the preferences of left-handers. For instance, as indicated earlier, 1.6% of people writing with their right hand prefer throwing left, while 28.8% of people writing left throw right [10]. The other important objective is to document the case of women because there are sex differences in the field of handedness [8] and footedness [9].

## Method

### Participants

Because there are many more right-handers than left-handers in the population [1–3], we were expecting participants to be mostly right-handed. We arbitrarily predetermined to stop recruitment once the sample reached 100 female and 100 male left-handed participants, based on the writing or throwing hand preference, whichever was attained first. Emphasizing writing and throwing was motivated by the impression that people often refer to these unimanual tasks to decide whether they are left- or right-handers [4].

A total of 854 people filled out the questionnaire between August 2020 and August 2022: 425 females (*M*_age_ = 33.1, S.D. = 15.3), 422 males (*M*_age_ = 34.8, S.D. = 17.0), and 7 non-binary people (*M*_age_ = 31.4, S.D. = 7.6). The average age for the whole sample was 33.9 years old (SD = 16.1; Range: 17.5–90.6). There was no age difference between the three groups (Kruskall-Wallis), *H*(2) = .241, *p* = .887. Sixty-four participants (62 males and two females) played in a recognized ice-hockey league. Out of 854 participants, 805 were Canadians (94.3%), 25 (2.9%) were from France, and 24 were from 16 different other countries.

### Measures

In addition to a four-item socio-demographic questionnaire (date of birth, gender, nationality, playing ice-hockey or not), four sets of questions were used: (1) unimanual handedness, (2) footedness, (3) eyedness, and (4) bilateral tasks (for a complete report of the questions related to laterality, see S1 Appendix). Note that all the variables were transformed in categorical variables because the main dependent variable (i.e., the way one holds an ice-hockey stick) was categorical and this encouraged consistency across analyses.

Handedness was measured with the *Edinburgh Handedness Inventory–Short Form* (EHI, [25]). Participants had to say which hand they used to execute four different unimanual daily tasks (writing, throwing, toothbrushing, and use of a spoon) on a 5-point scale from -100 “Always left” to 100 “Always right”. The average score of the four items was then used to assess handedness according to Veale’s classification, with average scores of -100 to -61 classified as left-handers, -60 to 60 as ambidextrous, and 61–100 as right-handers.

The sets of questions to assess footedness and eyedness were both drawn from Coren [26]. They both required participants to say which foot/eye they typically use in four different tasks (footedness: kick a ball, pick up a pebble with your feet, stomp on a bug and step onto a chair; eyedness: look through a telescope, look in a dark bottle, peep through a keyhole and aiming with a gun). Participants could answer “Left” (-1), “Right” (1) or “Either” (0). As in Coren’s [26] study, the sum of their answers represented their footedness/eyedness (i.e., ranging from -4 “completely left”, to 4 “completely right”, with 0 meaning “ambidextrous”). The sum was then categorized to allow consistency in the analyses, with participants with a sum from -4 to -1 being considered left-footed/left-eyed, and those with a sum from 1 to 4 being considered right-footed/right-eyed. Participants with a total score of 0 were considered ambidextrous.

Seven questions addressed bimanual tasks. In addition to a question about holding an ice-hockey stick, two other questions were related to other bimanual asymmetric sport activities: using a golf club, and using a baseball bat. The other four questions were related to non-sport activities: using a rake, a shovel, a broom, and an ax. For each of these seven questions, “1” indicated the placement of the left hand at the extremity of the handle, “2” meant no preference and “3”, the right hand at the extremity.

Because ice-hockey is central in the present analysis, it is important to keep in mind that shooting left means having the left hand near the middle of the stick and the right hand at the higher extremity of the stick; and shooting right means having the right hand near the middle of the stick and the left hand at the higher extremity of the stick. In other words, for a slap shot or wrist shot, the puck is at the left of a person shooting left, and at the right of a person shooting right.

### Procedure

Participants were recruited mainly via the mailing list of Université Laval (students and staff), but also via social media, an announcement on a national radio at the end of an interview of the first author on laterality, the mailing list of the *Society for International Hockey Research*, the mailing list of the *Société Québécoise pour la recherche en psychologie*, contact with college ice-hockey teams, and by word of mouth. The 23 questions were available online on the Limesurvey platform. Anyone from the general population could respond. Participants first had to read a consent form and agree to participate, and then respond to all the laterality questions (approx. 10 minutes). Participants were then invited (not mandatory) to provide contact information (e.g., email address) to enter into a draw for one of 40 $50-shopping cards at a bookstore. The responses given in the questionnaire were stored separately from the individual participants’ personal data.

### Analyses

Statistical analyses were ran using IBM SPSS, version 29. Frequencies and percentages of the different lateral preferences (handedness, eyedness, footedness, bilateral activities) are presented as a function of preferences when using an ice-hockey stick. Then, for measuring the degree of association between the lateral preference in ice-hockey (shooting left or right) and other categorical (prefer left, right or either) variables (handedness, eyedness, footedness), we used Cramér’s V or Phi (other bilateral tasks). Results were considered significant when *p* < .05.

## Results

### Distribution of preferences

#### Unimanual task preferences (handedness)

For the unilateral tasks, out of 854 participants, there are 583 (68.3%) right-handers, 140 (16.4%) ambidextrous, and 131 (15.3%) left-handers. Out of the 583 right-handers, 386 (66.2%) reported a preference for shooting left in ice-hockey, and 183 (31.4%) for shooting right (remaining participants reported shooting both ways; for all subsequent results, remaining participants will be those who reported shooting both ways). Out of the 131 left-handers, 34 (26.0%) reported a preference for shooting left in ice-hockey, and 92 (70.2%) for shooting right. As for the 140 ambidextrous, 43 (30.7%) reported a preference for shooting left in ice-hockey, and 86 (61.3%) for shooting right.

Table 1 summarizes the distribution of preferences for holding an ice-hockey stick according to the preferred hand for unilateral tasks. The information is reported for both male and female participants. Non-binary people were excluded from further analyses because of the low sample size (*n* = 7).

**Table 1:** Frequencies (and percentages) for unimanual tasks and for ice-hockey for male and female participants.

	Unimanual tasks (handedness)		Ice-hockey	
Male (*n* = 422)	Left	59 (14.0%)	Shoots left	14 (23.7%)
			Shoots both ways	1 (1.7%)
			Shoots right	44 (74.6%)
	Ambidextrous	78 (18.5%)	Shoots left	24 (30.8%)
			Shoots both ways	3 (3.8%)
			Shoots right	51 (65.4%)
	Right	285 (67.5%)	Shoots left	200 (70.2%)
			Shoots both ways	2 (0.7%)
			Shoots right	83 (29.1%)
Female (*n* = 425)	Left	71 (16.7%)	Shoots left	20 (28.2%)
			Shoots both ways	3 (4.2%)
			Shoots right	48 (67.6%)
	Ambidextrous	61 (14.4%)	Shoots left	18 (29.5%)
			Shoots both ways	8 (13.1%)
			Shoots right	35 (57.4%)
	Right	293 (68.9%)	Shoots left	184 (62.8%)
			Shoots both ways	12 (4.1%)
			Shoots right	97 (33.1%)

*Note*. The recruitment criterion of 100 left-handed males and females does not appear fulfilled in the current table. However, recruitment was stopped when 100 people of each gender reported a preference for either writing or throwing with their left hand. Some of them were later classified as ambidextrous because they were (for example) writing with their left, but eating with a spoon with their right.

#### Footedness

Out of the 854 participants reporting a foot preference, 677 (79.3%) prefer the right foot and 126 (14.8%) the left; 51 (6.0%) were ambidextrous. Out of the 677 people with a preference for the right foot, 401 (59.2%) prefer shooting left in ice-hockey and 255 (37.7%) prefer shooting right. Out of the 126 people with a preference for the left foot, 40 (31.7%) prefer shooting left and 81 (64.3%) prefer shooting right. As for the 51 ambidextrous, 22 (43.1%) reported a preference for shooting left in ice-hockey, and 25 (49.0%) for shooting right.

Table 2 summarizes the distribution of preferences for holding an ice-hockey stick according to the preferred foot. The information is reported for both male and female participants.

**Table 2:** Frequencies (and percentages) for footedness and for ice-hockey for male and female participants.

	Footedness		Ice-hockey	
Male (*n* = 422)	Left	59 (14.0%)	Shoots left	18 (30.5%)
			Shoot both ways	1 (1.7%)
			Shoots right	40 (67.8%)
	Ambidextrous	31 (7.3%)	Shoots left	17 (54.8%)
			Shoot both ways	1 (3.2%)
			Shoots right	13 (41.9%)
	Right	332 (78.7%)	Shoots left	203 (61.1%)
			Shoot both ways	4 (1.2%)
			Shoots right	125 (37.7%)
Female (*n* = 425)	Left	65 (15.3%)	Shoots left	21 (32.3%)
			Shoot both ways	3 (4.6%)
			Shoots right	41 (63,1%)
	Ambidextrous	19 (4.5%)	Shoots left	5 (26.3%)
			Shoot both ways	3 (15.8%)
			Shoots right	11 (57.9%)
	Right	341 (80.2%)	Shoots left	196 (57.5%)
			Shoot both ways	17 (5.0%)
			Shoots right	128 (37.5%)

#### Eyedness

Out of the 854 participants reporting an eye preference, 542 (63.5%) prefer the right eye and 258 (30.2%), the left; 54 (6.3%) are ambidextrous. Out of the 542 people with a preference for the right eye, 323 (59.6%) prefer shooting left in ice-hockey and 202 (37.3%) prefer shooting right. Out of the 258 people with a preference for the left eye, 115 (44.6%) prefer shooting left and 135 (52.3%) prefer shooting right. As for the 54 ambidextrous, 25 (46.3%) reported a preference for shooting left in ice-hockey, and 24 (44.4%) for shooting right.

Table 3 summarizes the distribution of preferences for holding an ice-hockey stick according to the preferred eye for both male and female participants.

**Table 3:** Frequencies (and percentages) for eyedness and for ice-hockey for male and female participants.

	Eyedness		Ice-hockey	
Male (*n* = 422)	Left	125 (29.6%)	Shoots left	53 (42.4%)
			Shoot both ways	3 (2.4%)
			Shoots right	69 (55.2%)
	Ambidextrous	26 (6.2%)	Shoots left	13 (50.0%)
			Shoot both ways	1 (3.8%)
			Shoots right	12 (46.2%)
	Right	271 (64.2%)	Shoots left	172 (63.5%)
			Shoot both ways	2 (0.7%)
			Shoots right	97 (35.8%)
Female (*n* = 425)	Left	130 (30.6%)	Shoots left	61 (46.9%)
			Shoot both ways	4 (3.1%)
			Shoots right	65 (50.0%)
	Ambidextrous	27 (6.4%)	Shoots left	12 (44.4%)
			Shoot both ways	4 (14.8%)
			Shoots right	11 (40.7%)
	Right	268 (63.1%)	Shoots left	149 (55.6%)
			Shoot both ways	15 (5.6%)
			Shoots right	104 (38.8%)

#### Bimanual task preferences

*Sport activities*. Out of the 811 participants reporting a preference when swinging a golf club, 445 (52.1%) prefer to swing like a right-hander (in the golf terminology; left hand at the extremity) and 366 (42.9%) like a left-hander. Out of the 445 people with a preference for swinging right, 103 (23.1%) prefer shooting left in ice-hockey and 333 (74.8%) prefer shooting right. Out of the 366 people with a preference for swinging left, 342 (93.4%) prefer shooting left in ice-hockey and 19 prefer shooting right (5.2%).

Out of the 811 participants reporting a preference when swinging a baseball bat, 309 (36.2%) prefer batting right (left hand at the extremity) and 502 (58.8%) batting left. Out of the 309 people with a preference for batting right, 265 (85.8%) prefer shooting left in ice-hockey and 37 (12.0%) prefer shooting right. Out of the 502 people with a preference for batting left, 171 (34.1%) prefer shooting left in ice-hockey and 316 (62.9%) prefer shooting right.

Table 4 summarizes the distributions of preferences for holding an ice-hockey stick according to the placement of hands when playing golf or batting in baseball for both male and female participants.

**Table 4:** Frequencies (and percentages) of lateral preferences in ice-hockey as a function of lateral preferences for golf, for swinging a baseball bat, for using an ax, a shovel, a broom, and a rake.

	**Golf**		Ice-hockey	
Male (*n* = 422)	Left	180 (42.7%)	Shoots left	172 (95.6%)
			Shoots right	8 (4.4%)
	Right	226 (53.6%)	Shoots left	57 (25.2%)
			Shoots right	169 (74.8%)
Female (*n* = 425)	Left	178 (41.9%)	Shoots left	167 (93.8%)
			Shoots right	11 (6.2%)
	Right	207 (48.7%)	Shoots left	46 (22.2%)
			Shoots right	161 (77.8%)
	**Baseball**		Ice-hockey	
Male (*n* = 422)	Left	228 (57.3%)	Shoots left	71 (31.1%)
			Shoots right	157 (68.9%)
	Right	170 (42.7%)	Shoots left	154 (90.6%)
			Shoots right	16 (9.4%)
Female (*n* = 425)	Left	256 (66.5%)	Shoots left	99 (38.7%)
			Shoots right	157 (61.3%)
	Right	129 (33.5%)	Shoots left	109 (84.5%)
			Shoots right	20 (15.5%)
	**Ax**		Ice-hockey	
Male (*n* = 422)	Left	229 (58.0%)	Shoots left	67 (29.3%)
			Shoots right	162 (70.7%)
	Right	166 (42.0%)	Shoots left	156 (94.0%)
			Shoots right	10 (6.0%)
Female (*n* = 425)	Left	253 (66.4%)	Shoots left	105 (41.5%)
			Shoots right	148 (58.5%)
	Right	128 (33.6%)	Shoots left	106 (82.8%)
			Shoots right	22 (17.2%)
	**Shovel**		Ice-hockey	
Male (*n* = 422)	Left	227 (60.5%)	Shoots left	215 (94.7%)
			Shoots right	12 (5.3%)
	Right	148 (39.5%)	Shoots left	4 (2.7%)
			Shoots right	144 (97.3%)
Female (*n* = 425)	Left	209 (61.7%)	Shoots left	173 (82.8%)
			Shoots right	36 (17.2%)
	Right	130 (38.4%)	Shoots left	16 (12.3%)
			Shoots right	114 (87.7%)
	**Broom**		Ice-hockey	
Male (*n* = 422)	Left	207 (59.7%)	Shoots left	198 (95.7%)
			Shoots right	9 (4.4%)
	Right	140 (40.4%)	Shoots left	6 (4.3%)
			Shoots right	134 (95.7%)
Female (*n* = 425)	Left	187 (59.9%)	Shoots left	163 (87.2%)
			Shoots right	24 (12.8%)
	Right	125 (40.1%)	Shoots left	16 (12.8%)
			Shoots right	109 (87.2%)
	**Rake**		Ice-hockey	
Male (*n* = 422)	Left	218 (60.2%)	Shoots left	208 (95.4%)
			Shoots right	10 (4.6%)
	Right	144 (39.8%)	Shoots left	6 (4.2%)
			Shoots right	138 (95.8%)
Female (*n* = 425)	Left	197 (61.2%)	Shoots left	166 (84.3%)
			Shoots right	31 (15.7%)
	Right	125 (38.8%)	Shoots left	17 (13.6%)
			Shoots right	108 (86.4%)

*Non sport activities*. Out of the 803 participants reporting a preference when using an ax, 306 (35.8%) prefer swinging right (left hand at the extremity) and 497 (58.2%) prefer swinging left. Out of the 306 people with a preference for swinging right, 264 (86.3%) prefer shooting left in ice-hockey and 33 (10.8%) prefer shooting right. Out of the 497 people with a preference for swinging left, 173 (34.8%) prefer shooting left in ice-hockey and 312 prefer shooting right (62.8%).

As for the other three bilateral non sport activities (i.e., shovel, broom, rake), they essentially exhibit the same pattern. The preference in one of these activities is a good predictor of the position of the hands when holding an ice-hockey stick (uncrossed preference) and this prediction in all three cases is descriptively stronger for male than for female participants. Table 4 illustrates these similar trends across bilateral tasks.

### Strength of associations

The strength of associations between the lateral preference in ice-hockey (shooting left or right) and other dichotomic variables, for male and female participants, and for the overall sample are reported in Table 5. All Cramer’s Vs (for unimanual tasks, footedness and eyedness) and Phi (for bilateral tasks) are significant (*p* < .001), except the relation between the placement of hands in ice-hockey and eyedness of female participants.

**Table 5:** Strength of associations (Cramer’s V and Phi) between the lateral preference in ice-hockey and other categorical variables, for male and female participants, and for the overall sample.

	Overall	Male	Female
Handedness	**.353**	**.399**	**.315**
Footedness	**.205**	**.215**	**.216**
Eyedness	**.144**	**.188**	**.098**
Golf	.713	.705	.718
Baseball	**.515**	**.593**	**.434**
Rake	.808	.908	.695
Shovel	.806	.912	.690
Broom	.828	.911	.737
Ax	**.520**	**.644**	**.393**

*Note*. Bold indicates cross-lateral (left-right or right-left) preferences, i.e., “reverse stance” cases.

## Discussion

The study reveals that most people reporting being right-handers prefer shooting left in ice-hockey and that most people reporting being left-handers prefer shooting right; this tendency is descriptively stronger amongst male than female participants. Amongst people ambidextrous for unilateral tasks, there was a clear preference for shooting right. Most participants also showed a right foot and a right eye preference, and most showed foot-ice-hockey and eye-ice-hockey cross-lateral preference. As for bilateral asymmetrical tasks, there is uncrossed lateral preference for ice-hockey and each of four tools, namely broom, shovel, rake, and golf club, but cross-lateral preference with baseball bat and ax.

The main finding of the study is the fact that most participants have a handedness-ice-hockey cross-lateral preference. This finding is not totally surprising given that it is consistent with the few studies available on this topic [19, 23] and the fact that in North American professional ice-hockey, there is traditionally more ice-hockey players shooting left than right [21, 27]. However, these data are inconsistent with the main report (largest sample), by Loffing et al., showing data for handedness and lateral preference in ice-hockey [13]: 591 out of their 812 participants reporting a preference when holding a hockey stick choose to shoot right (see their Table S2). It cannot be excluded that this inconsistency is due to the different levels of exposure to ice-hockey, and therefore the amount of learning by observations, familiarity of German vs. Canadian participants.

On a side note, cultural differences may not explain the whole discrepancy. Data collected amongst ice-hockey players over the history of the NHL reveal that relatives (fathers and sons, uncles, grandchildren) are slightly more likely to shoot from the same side, which would mean that there is a genetic component for explaining laterality preference in ice-hockey that go beyond familiarity or cultural differences [27]. Still, the percentages of relatives shooting from the same side are not closely linked to their degree of relatedness (i.e., closer relatives are not more likely to shoot from the same side than more distant relatives). This means that an environmental (cultural) component may be at play in the lateral preference in ice-hockey. However, the environmental explanation also requires nuances. Indeed, the same data set [27] indicate that the years following the emergence of superstars were not aligned with the adoption of the same lateral preference as said superstars (e.g., players growing up watching and admiring Wayne Gretzky who was shooting from the left were not more likely to shoot from the left).

A problem however about the environmental hypothesis (or learning by observation issue), when applied to the German case, is that, although unfamiliar, this preference for shooting right is not apparent in the higher levels of German hockey. For example, Germany was the finalist in the 2023 ice-hockey World Championship. Out of 22 players (forwards or defense), 15 were shooting left. That is to say that, amongst people in Germany familiar with ice-hockey, they are more likely to shoot left than the general German population. Clearly, there is room here for additional investigations based on cultural differences, but also some need for additional control within countries. Apparently, the understanding of the requirements of the game and skill level might be a critical factor. If a participant unfamiliar with ice-hockey is asked to hold an ice-hockey stick and to shoot with it, adopting a stance with their dominant hand in the middle makes sense (as in baseball) as it would favour power [17, 19]. But if control and dribbling with the puck is emphasised, then placement of hands might need to be changed to the opposite.

### Strength of associations between ice-hockey and other lateral preferences

For footedness and eyedness, the link with lateral preference for holding an ice-hockey stick is quite low, and in both cases, indicates cross preferences (left-right or right-left). The proportion of left-footed participant, approximately 15%, is consistent with the reports in the literature, but males are not more likely than females to be left-footed in the present study [9]. For eyedness, about 30% of people in our sample (about the same proportions for female and male participants) had a left-eye preference, which is consistent with what is reported in the literature [10]. The weak link between eyedness and gripping an ice-hockey stick is not surprising considering that in bilateral asymmetric sports like cricket and golf, the advantage of a cross-lateral stance is not modulated by eyedness [14, 15] In the review by Moreno et al. [28], cross hand-eye lateral preference would be helpful in tennis and some team sports, and hockey is mentioned in these “team sports”. However, it is not specified if it is ice-hockey or field hockey, and what belongs specifically to hockey; we have not found this information in the original paper [29].

The associations of hand placement on an ice-hockey stick with other bilateral asymmetrical tasks are interesting. Three non-sport activities (broom, shovel, and rake) led to very high, and uncrossed, association. The association with the other non-sport activity, using an ax, was also high, but crossed. This last activity differs from the other three for requiring a lot of power, and for the reduced distance between hands. As for the sport activities (i.e., baseball and golf), their association with lateral preference in ice-hockey was high, especially for golf, but direction of the links was not the same: uncrossed for golf and crossed for baseball. In this case, it is not the distance between hands that seems to be at play. Indeed, the number of participants batting left in baseball is very high in our sample, compared to the proportion met in Major League Baseball [17]; this could be partly explained by the higher proportion of left-handers in our sample. As well, the number of participants adopting a left position for golf is amazingly high, but contrary to the baseball case, most people playing right in golf will shoot right in ice-hockey, and almost 95% of those playing left in golf will play left in ice-hockey. A tentative explanation for this strong correlation between the golf and ice-hockey lateral preference lies on the possibility that Canadians learn ice-hockey first and transfer their lateral skill (habit) to golf (there is a high number of left-handers in our sample, which may be explained by the sensitivity of this population to our search for participants in a study about laterality). Indeed, it is known that the practice of certain asymmetric bimanual sports activities affects performance in other activities that require the participation of each hand. For example, the practice of hockey among Canadians influences their baseball performance [30, 31].

### Limitations

The present investigation suffers from some noticeable limitations. One is the fact that it was conducted on-line. This means that if participants had questions, or uncertainties about the tasks, they received no response. One question could have been for example whether the task related to golf was for driving or for putting. Another limitation is related to the provenance of participants mainly from Canada and, more specifically, mainly from Québec. This means that participants were likely familiar with ice-hockey and exposed to the way of gripping the stick, and probably knew about the need to dribble and not only about the need to shoot.

The way handedness, eyedness, and footedness was tested is also a limitation. For example, there are different ways of assessing eye preference or dominance [28], including using photo and a pointing task [15], instead of using preferences for a one-eyed activity. As for the bilateral tasks, it is not excluded that the order of questions may have had an impact, with the response to one question having repercussion on the way of representing the placement of hands on the subsequent question.

Finally, even if unlikely, there is a possibility that some participants took part more than once in the study, which would artificially inflate the observed associations. No single participant reported the exact same set of answers (including their contact information to participate in the gift-card draw).

### Future investigations

There are still many avenues to explore to understand what the main factors are in determining lateral preference in ice-hockey and what is at stake in this hitting /control trade-off. This field of investigation would probably gain additional insights from studies dedicated to other sports that also require asymmetric contributions of the hands. Sports like hurling in Ireland, or lacrosse in North America, are interesting cases because of their specific requirements when holding the bat or the stick, and the need to move on the field. In addition to a survey on the placement of hands in these sports by experts and novices, information could be drawn, at least for ice-hockey or lacrosse, from experiments where power and control (with different tests), could be compared when hands are placed in the preferred vs. nonpreferred position.

Additional information would likely be gained from another sensory preference: the vestibular preference. There is indeed not much information about the preference for rotating in one direction or another along a vertical axis [32] in the context of ice-hockey. Loffing et al. [13] asked their participants in which direction they would prefer rotating in figure skating and report a very poor relationship with handedness. However, tasks requiring the contribution of one hand might not require as much rotation as shooting in ice-hockey, for example. In addition, playing ice-hockey often requires turning in both clockwise and counterclockwise directions, skills that are not necessarily mastered equally well by players. It is not possible to exclude the hypothesis of a connection between a preference for rotating and the way of holding a hockey stick. What is required is to establish the link between the preference that could be observed in a test like the Fukuda’s stepping test [33], for example, and the preferences for hand placements in asymmetrical tasks that also involve rotations. But what would remain difficult to determine, as noted by Heinen et al. [32], is whether such a rotational preference would cause other processing preferences, or would be an effect of it.

The question addressing how lateral preference in bimanual asymmetrical tasks is determined during the development is also widely unaddressed in the literature and could be crucial for understanding why one shoots left or right in ice-hockey [27, 34]. In short, much remains to be discovered in the potential links between overt lateralized behaviors and the covert brain functioning that determines lateral preferences.

### Implications

From a practical standpoint, the particularities of ice-hockey (lateral requirements of the game and grasping of the stick) are such that lateral issues would probably be a relevant variable to consider in talent identification. While most left-wingers and right-wingers typically shot left and right, respectively, there are instances of famous ice-hockey left wingers (like Russians V. Kamenski and A. Ovechkin) shooting right and right-wingers shooting left (like Canadians M. Richard and Y. Cournoyer) (i.e., ‘off-wing’ wingers). Indeed, amongst left wingers, those who shoot right tend to accumulate more points than those who shoot left, and this type of effect applies to right wingers, with those shooting left accumulating more points than those shooting right [35] (it cannot be excluded that the success of left-wingers shooting right and right-wingers shooting left be due to some perceptual frequency effect comparable to the one providing advantage to left-handers in tennis [36] and volleyball [37], for example). This could, for example, help scouts and coaches make selections when they are hesitating between players, favoring ‘off-wing’ wingers. We know that talent identification in ice-hockey could be partly based on perceptual-cognitive variables skills (eye movements and decision making) and self-regulation [38], but adding systematic testing of handedness and eyedness, in addition to the way of grasping the stick, would likely be helpful. At least, considering handedness, in addition to the bimanual preference, and assessing eye preference or dominance, would likely help coaches to guide the development of players more efficiently. If coaches are aware of a player’s preferred eye or hand and observe a (mis)fit with their lateral preference in ice-hockey, they can adapt said player’s role more efficiently. For example, they might try switching a left winger to the right to see if their abilities are better utilized there.

## Conclusion

According to Guiard, considering that frame of reference provided by the nonpreferred hand that acts first (guides the motion executed by the preferred hand), the nonpreferred hand should be placed above the preferred hand in activities like in golf or baseball requiring single-swing outcomes and power [11, 22]. The present study reveals that a majority of people do not adopt such a stance in ice-hockey. In addition to shooting (swinging), ice-hockey requires stick handling. By the adoption a cross-lateral preference, people invest their preferred hand in a role requiring movements of lesser magnitude during stick handling.

The gap between the results of the present study and those of Loffing et al. [13] about ice-hockey indicates that this sport discipline is an interesting tool for exploring properties of human laterality. This gap reveals the need to be precise about the purpose of the task when assessing the lateral preference of some asymmetrical bimanual tasks. For ice-hockey, when testing participants, it would be necessary to specify if the purpose is shooting (or slapping) or handling the puck. Because of the close link observed between the lateral preference in ice-hockey and golf in the present study, it would likely be well-advised to clarify, when assessing the lateral preference for golf, whether the golf task requires putting (which requires no power) or driving (where exerting power through the swing could be searched.

## Supporting information

S1 Appendix List of laterality-related questions asked to participants. (DOCX)

S1 Checklist STROBE statement—Checklist of items that should be included in reports of observational studies. (PDF)
