# Supplementary material for: Linking the preference in a bilateral asymmetric task with handedness, footedness, and eyedness: The case of ice-hockey
Source: PLoS One. 2024 May 23;19(5):e0294125. doi: 10.1371/journal.pone.0294125 (PMC11115253; doi:10.1371/journal.pone.0294125)
Supplement: S1 Appendix — (DOCX) [file pone.0294125.s001.docx]

**Appendix A**

**List of laterality-related questions asked to participants**

Data are publicly available here: https://osf.io/h95rv/?view_only=d6d1a10951f04c37b26cde5ad69f5082

One-handed task questions

Please indicate your preferences in the use of hands in the following activities or use of object.

|  | **Always left (-100)** | **Usually left (-50)** | **Both equally (0)** | **Usually right (50)** | **Always right (100)** |
| --- | --- | --- | --- | --- | --- |
| Writing |  |  | 2 (0.2%) |  |  |
| Throwing |  |  | 15 (1.8%) |  |  |
| Toothbrush |  |  | 40 (4.7%) |  |  |
| Spoon |  |  | 78 (9.1%) |  |  |

Footedness-related questions

Simply read each of the questions below. Decide which foot you use for each activity and then select the answer that describes you the best. If you are unsure of any answer, try to act out the action.

|  | **Left** | **Either** | **Right** |
| --- | --- | --- | --- |
| With which foot would you kick a ball to hit a target? |  | 50 (5.9%) |  |
| If you wanted to pick up a pebble with your toes, which foot would you use? |  | 324 (37.9%) |  |
| Which foot would you use to step on a bug? |  | 397 (46.5%) |  |
| If you had to step up onto a chair, which foot would you place on the chair first? |  | 239 (28.0%) |  |

Eyedness-related questions

Simply read each of the questions below. Decide which eye you use for each activity and then put a check mark next to the answer that describes you the best. If you are unsure of any answer, try to act out the action.

|  | **Left** | **Either** | **Right** |
| --- | --- | --- | --- |
| Which eye would you use to look through a telescope? |  | 129 (15.1%) |  |
| If you had to look into a dark bottle to see how full it was, which eye would you use? |  | 156 (18.3%) |  |
| Which eye would you use to peep through a keyhole? |  | 116 (13.6%) |  |
| Which eye would you use to sight down a rifle? |  | 42 (4.9%) |  |

Two-handed task questions

For the following questions, please indicate how you place both hands when using the following objects.

|  | **Left hand at the end and right hand lower** | **Both ways without preference** | **Right hand at the end and left hand lower** |
| --- | --- | --- | --- |
| A hockey stick |  | 30 **(**3.5%) |  |
| A golf club |  | 43 (5.0%) |  |
| A rake |  | 158 (18.5%) |  |
| A shovel |  | 125 (14.6%) |  |
| A broom or a mop |  | 185 (21.7%) |  |
|  | **Left hand at the end and right hand higher** | **Both ways without preference** | **Right hand at the end and left hand higher** |
| A baseball bat |  | 43 (5.0%) |  |
| An ax |  | 51 (6.0%) |  |
