# Supplementary material for: Linking the preference in a bilateral asymmetric task with handedness, footedness, and eyedness: The case of ice-hockey
Source: PLoS One. 2024 May 23;19(5):e0294125. doi: 10.1371/journal.pone.0294125 (PMC11115253; doi:10.1371/journal.pone.0294125)
Supplement: S1 Checklist — (PDF) [file pone.0294125.s002.pdf]

STROBE Statement—checklist of items that should be included in reports of observational studies

| | Item No. | Recommendation | Page No. | Relevant text from manuscript |
|---|---|---|---|---|
| **Title and abstract** | 1 | (*a*) Indicate the study's design with a commonly used term in the title or the abstract | 2 | In this study (*n* = 854), the main objective was to determine to what extent the manner of holding a hockey stick could be predicted based on other lateralized preferences. |
| | | (*b*) Provide in the abstract an informative and balanced summary of what was done and what was found | 2 | Amongst the 201 participants reporting a preference for writing with the left hand, 77.6% reported a preference for shooting right (placing the right hand in the middle of the stick); and amongst the 621 participants reporting a preference for writing with the right hand, 67,0% reported a preference for shooting left. This preference on hockey stick is closely related to the way one holds a rake, shovel, or broom ($rs_{pb}$ > .80), or a golf club ($r_{pb}$ = .72), but inversely related to the way one holds an ax ($r_{pb}$ = -.52), a baseball bat |

| | | | | |
|---|---|---|---|---|
| | | | | ($r_{pb}$ = -.52), or when doing unimanual tasks ($rs_{pb}$ between -.20 and -.39). There is no link between the way of holding the hockey stick and ocular ($r_{pb}$ = .07) or footedness preferences ($r_{pb}$ = .02). |
| **Introduction** | | | | |
| Background/rationale | 2 | Explain the scientific background and rationale for the investigation being reported | 3-5 | Most people can tell their left from their right and know, almost spontaneously, whether they are left-handed or right-handed. Several daily activities require the contribution of only one hand (for example, holding a spoon while eating). This spontaneous knowledge about our own handedness very likely reveals that our common representation of handedness is based on the use of hands in daily unimanual activities. The predominant use of one hand over the other in such daily activities is a good predictor of which hand a person will use to practice a single-handed tool-manipulation sport (for example, using a racket in table tennis, tennis, or |

badminton; [1]). However, the prediction of the preferred foot for sports centered on jump (long or high) or skateboarding is poorly predicted by handedness [1]. Even within the scope of manual activities, there are cases where preferences are poorly predicted by handedness: these cases are susceptible to occur when these activities require the contribution of both hands (bimanual activities), and, more specifically, when the hands do not have the same role, as is the case when using an ax, for example [2].

The present study will use ice hockey to analyze hand preferences in uni- and bimanual tasks. This sport is most interesting in the study of laterality as the hands clearly play different, asymmetric, roles. Ice hockey is even more interesting from a lateral viewpoint since the organization of the game itself presents a lateral component (having left or right wingers, left or right defenders); the combination of the position occupied on the ice

rink and the way of holding the hockey stick therefore results in different constraints or possibilities [3].

Contrary to activities such as batting in baseball or swinging with a golf club, the position of hands on the hockey stick are not close to each other, as they do not touch each other. Ice hockey should not be confused with field hockey where the stick is much shorter and each player shoots from the right. Ice hockey is particularly fascinating for having to accommodate the need for shooting with power, as well as the need for control and finesse in stick handling; both functions command a different hand placement on the stick [4]. Therefore, ice hockey is an interesting tool for exploring properties of human laterality from a unique angle.

In bimanual tasks where the two hands are assigned different roles, there are principles of cooperation between the hands [2]. According to one principle, the dominant hand (manual

preference) is more likely to be assigned to a portion of the task requiring fine resolution (spatial or temporal), with the nondominant hand playing a supporting role [2]. In ice hockey, manual preference does not determine lateral preference very accurately, namely the way of holding the stick [4]. In fact, we know little about the interaction between manual preferences and the way of grasping the hockey stick. Traditionally, in ice hockey, more players shoot left (left hand near the middle of the stick) than right (right hand near the middle of the stick). For example, the percentage of left-shooters in the NHL increased from 57% between the years 1917–1926 to 67% between 1997 and 2006, and varies slightly depending on position, forwards versus defensemen [5]. The percentage of goalies who catch from the left increased from 88 in years 1957-1966 to 91 in years 1997-2006 [5]. Only a few studies are available on the links between hand

preference and lateral preference with a hockey stick, and the link appears to be weak. In a study of 194 male hockey players, 55.6% of those with a left-hand preference (n = 27) were shooting right (right hand in the middle of the stick, with the stick at the right of the shooter), and 67.1% of those with a right-hand preference (n = 167) were shooting left [4]. In a survey of 214 men in the general population, 65.7% of left-handers were shooting right and 55.9% of 179 right-handers were shooting left [6].

In a more recent study of a German sample of 812 participants though, Loffing and colleagues [1] found that the correlation (point biserial) between the preferred hand in a unilateral task (based on the German version of the Edinburgh Handedness Inventory) and the position of hands on the ice hockey stick was .254, and this link was stronger for women (.328) than for men (.175). What is most surprising in their study is the

fact that out of 327 male right-handers, 249 (76,15%) shot right, and out of 409 female right-handers, 313 (76,53%) shot right. These data contradict what is reported in the previous literature, a fact that might be explained by the non-familiarity of German participants with the requirements in ice hockey. Amongst the left-handers in Loffing et al. [1], 50% out of 38 males and 73,68% out of 38 females were shot left.

Finally, we know that the practice of certain asymmetric bimanual sports activities affects performance in other activities that require the participation of each hand. For example, the practice of hockey among Canadians influences their baseball performance [7],[8] . Also, the left-right distribution of eye preference, with about f people having a left eye preference, is less marked than that for hand preference, and that a match of eye and hand preferences may, for example, grant an advantage in sports, at least in the case of

| | | | | |
|---|---|---|---|---|
| | | | | duels [9]. |
| Objectives | 3 | State specific objectives, including any prespecified hypotheses | 6 | The general objective of this study is to determine to what extent the preferred way of holding a hockey stick could be predicted based on other lateralized preferences. More specifically, we will look at how the way of grasping an ice hockey stick is linked to other uni- and bimanual tasks, to footedness, and to ocular preferences. The bimanual tasks of interest are those involving an asymmetric contribution of hands, with some requiring low power requirements (broom, rake, shovel) and other more power (ax, baseball, and golf). For all of the analyses, it is intended to determine if the overall observations apply equally to male and female participants. |
| **Methods** | | | | |
| Study design | 4 | Present key elements of study design early in the paper | 7-8 | Participants were recruited mainly via the mailing list of Université Laval (students and staff), social media, an announcement on a national radio at the end of an interview |

| | | | | |
|---|---|---|---|---|
| | | | | of the first author on laterality, the mailing list of the Society for International Hockey Research, the mailing list of the Société Québécoise pour la recherche en psychologie, contact with college hockey teams, and by word of mouth. The 23 questions were available online on the Limesurvey platform. Anyone from the general population could respond. Participants had first to read a consent form and agree to participate, and then take approximately 10 minutes to respond to all questions. Participants were then entered into a draw for one of 40 $50-shopping cards at a bookstore. Therefore, authors had access to information that could identify individual participants during or after data collection to be able to contact them if they won the draw. |
| Setting | 5 | Describe the setting, locations, and relevant dates, including periods of recruitment, exposure, follow-up, and data collection | 7-8 | Participants were recruited mainly via the mailing list of Université Laval (students and staff), social media, an announcement on a national radio at the end of an interview |

| | | | | |
|---|---|---|---|---|
| | | | | of the first author on laterality, the mailing list of the Society for International Hockey Research, the mailing list of the Société Québécoise pour la recherche en psychologie, contact with college hockey teams, and by word of mouth. The 23 questions were available online on the Limesurvey platform. Anyone from the general population could respond. Participants had first to read a consent form and agree to participate, and then take approximately 10 minutes to respond to all questions. Participants were then entered into a draw for one of 40 $50-shopping cards at a bookstore. Therefore, authors had access to information that could identify individual participants during or after data collection to be able to contact them if they won the draw. |
| Participants | 6 | (*a*) *Cohort study*—Give the eligibility criteria, and the sources and methods of selection of participants. Describe methods of follow-up<br>*Case-control study*—Give the eligibility criteria, and the sources and methods of case ascertainment and control selection. Give the rationale for the choice of cases and controls<br>*Cross-sectional study*—Give the eligibility criteria, and the sources and methods of selection of participants | 6 | Recruitment of participants was determined by evaluating handedness based on either writing or throwing preference. A predetermined target of 100 female and 100 male left- |

| | | | | |
|---|---|---|---|---|
| | | | | handed participants was reached based on the writing criterion. A total of 854 people filled out the questionnaire between August 2020 and August 2022: 425 females, 422 males, and 7 non-binary people, see Table 1. The average age was 33.9 years old (SD = 16.1; Range: 17.5 – 90.6). There was no age difference between the three groups, F(2, 853) = 1.353, p = .259, η = .003. Sixty-four participants (62 males) played in a recognized ice hockey league. Out of 854 participants, 805 are Canadians (94.4%), 25 (2.9%) are from France, and 24 are from 16 different other countries. |
| | | (*b*) *Cohort study*—For matched studies, give matching criteria and number of exposed and unexposed<br>*Case-control study*—For matched studies, give matching criteria and the number of controls per case | | |
| Variables | 7 | Clearly define all outcomes, exposures, predictors, potential confounders, and effect modifiers. Give diagnostic criteria, if applicable | 7 | In addition to a four-item socio-demographic questionnaire (birthdate, gender, nationality, playing ice hockey or not), three sets of questions were used, one for footedness, one for eyedness, and one for handedness based on two- |

| | | | | |
|---|---|---|---|---|
| | | | | handed tasks. Four questions from Veale [10] addressed unimanual tasks (writing, throwing, toothbrushing, and use of a spoon), with responses varying from 1, always left, to 5, always right.<br>Eight questions were drawn from Coren (1993) [11], four addressing eyedness and four addressing footedness, with responses from 1 (left) to 3 (right), 2 meaning both. The mean of the four items was kept for the analyses. A score below 2 was interpreted as a left preference, above 2 as a right preference, and 2 as no preference. Finally, seven questions were asked to address bimanual tasks: holding an ice hockey stick, a golf club, a baseball bat, a rake, a shovel, a broom, and an ax. For the bimanual tasks, 1 indicated using the right hand at the extremity and 3 the left hand, 2 meaning no preference. For a complete report of questions, see S1 Appendix A. |
| Data sources/ measurement | 8* | For each variable of interest, give sources of data and details of methods of assessment (measurement). Describe comparability of assessment methods if there is more than one group | 7 | In addition to a four-item socio-demographic questionnaire |

(birthdate, gender, nationality, playing ice hockey or not), three sets of questions were used, one for footedness, one for eyedness, and one for handedness based on two-handed tasks. Four questions from Veale [10] addressed unimanual tasks (writing, throwing, toothbrushing, and use of a spoon), with responses varying from 1, always left, to 5, always right.

Eight questions were drawn from Coren (1993) [11], four addressing eyedness and four addressing footedness, with responses from 1 (left) to 3 (right), 2 meaning both. The mean of the four items was kept for the analyses. A score below 2 was interpreted as a left preference, above 2 as a right preference, and 2 as no preference. Finally, seven questions were asked to address bimanual tasks: holding an ice hockey stick, a golf club, a baseball bat, a rake, a shovel, a broom, and an ax. For the bimanual tasks, 1 indicated using the right hand at the

| | | | | |
|---|---|---|---|---|
| | | | | extremity and 3 the left hand, 2 meaning no preference. For a complete report of questions, see S1 Appendix A. |
| Bias | 9 | Describe any efforts to address potential sources of bias | N/A | N/A |
| Study size | 10 | Explain how the study size was arrived at | 6 | Recruitment of participants was determined by evaluating handedness based on either writing or throwing preference. A predetermined target of 100 female and 100 male left-handed participants was reached based on the writing criterion. |

Continued on next page

| | | | | |
|---|---|---|---|---|
| Quantitative variables | 11 | Explain how quantitative variables were handled in the analyses. If applicable, describe which groupings were chosen and why | 8 | Analyses were conducted on IBM SPSS Statistics version 28. For the sake of simplicity, most analyses are based on a dichotomic approach. The number of cases for each question where people answered "I don't know" or "No preference" is reported in Appendix A.<br>A biserial-point correlation was calculated for quantifying the link between each of the lateralized tasks measured. Also, stepwise logistic regressions were conducted to predict the side a person shoots in ice hockey based on the hand with which they write, throw or a combination of both. |
| Statistical methods | 12 | (*a*) Describe all statistical methods, including those used to control for confounding | 8 | Analyses were conducted on IBM SPSS Statistics version 28. For the sake of simplicity, most analyses are based on a dichotomic approach. The number of cases for each question where people answered "I don't know" or "No preference" is reported in Appendix A.<br>A biserial-point correlation was calculated for quantifying the link between each of the lateralized tasks measured. Also, stepwise logistic regressions were conducted |

| | | | | |
|---|---|---|---|---|
| | | | | to predict the side a person shoots in ice hockey based on the hand with which they write, throw or a combination of both. |
| | | (*b*) Describe any methods used to examine subgroups and interactions | | |
| | | (*c*) Explain how missing data were addressed | N/A | |
| | | (*d*) *Cohort study*—If applicable, explain how loss to follow-up was addressed<br>*Case-control study*—If applicable, explain how matching of cases and controls was addressed<br>*Cross-sectional study*—If applicable, describe analytical methods taking account of sampling strategy | N/A | |
| | | (*e*) Describe any sensitivity analyses | N/A | |
| **Results** | | | | |
| Participants | 13* | (a) Report numbers of individuals at each stage of study—eg numbers potentially eligible, examined for eligibility, confirmed eligible, included in the study, completing follow-up, and analysed | N/A | |
| | | (b) Give reasons for non-participation at each stage | N/A | |
| | | (c) Consider use of a flow diagram | N/A | |
| Descriptive data | 14* | (a) Give characteristics of study participants (eg demographic, clinical, social) and information on exposures and potential confounders | 6 | A total of 854 people filled out the questionnaire between August 2020 and August 2022: 425 females, 422 males, and 7 non-binary people, see Table 1. The average age was 33.9 years old (SD = 16.1; Range: 17.5 – 90.6). There was no age difference between the three groups, $F(2, 853) = 1.353$, $p = .259$, $\eta = .003$. Sixty-four participants (62 males) played in a recognized ice hockey league. Out of 854 participants, 805 are Canadians (94.4%), 25 (2.9%) are from France, and 24 are from 16 different other countries. |

| | | | | |
|---|---|---|---|---|
| | | (b) Indicate number of participants with missing data for each variable of interest | N/A | |
| | | (c) *Cohort study*—Summarise follow-up time (eg, average and total amount) | N/A | |
| Outcome data | 15* | *Cohort study*—Report numbers of outcome events or summary measures over time | N/A | |
| | | *Case-control study*—Report numbers in each exposure category, or summary measures of exposure | N/A | |
| | | *Cross-sectional study*—Report numbers of outcome events or summary measures | 9 | A total of 854 people filled out the questionnaire between August 2020 and August 2022: 425 females, 422 males, and 7 non-binary people, see Table 1. The average age was 33.9 years old (SD = 16.1; Range: 17.5 – 90.6). There was no age difference between the three groups, $F(2, 853) = 1.353$, $p = .259$, $\eta = .003$. Sixty-four participants (62 males) played in a recognized ice hockey league. Out of 854 participants, 805 are Canadians (94.4%), 25 (2.9%) are from France, and 24 are from 16 different other countries. |
| Main results | 16 | (*a*) Give unadjusted estimates and, if applicable, confounder-adjusted estimates and their precision (eg, 95% confidence interval). Make clear which confounders were adjusted for and why they were included | 9-19 | Throwing<br>For the throwing task, out of 812 participants reporting a preference, 667 (82.14%) are right-handers and 145 (17.86%) left-handers. Out of the 667 right-handers, 407 (61.02%) reported a preference for shooting left in ice hockey, and 260 (38.98%) for shooting right. Out of the 145 left-handers, 51 (35.17%) reported a preference for shooting left in ice hockey, and 94 (64.83%) |

| | | | |
|---|---|---|---|
| | | | for shooting right.<br>Using a toothbrush<br>Out of the 790 participants reporting a preference when brushing their teeth, 185 (23.42%) prefer using the left hand, and 605 (76.58%) the right hand. Out of the 185 people with a preference for the left hand, 40 (21.62%) prefer shooting left in ice hockey and 145 (78.38%) prefer shooting right. Out of the 605 people with a preference for the right hand, 407 (67.27%) prefer shooting left in ice hockey and 198 prefer shooting right (32.73%).[…] |
| | (*b*) Report category boundaries when continuous variables were categorized | N/A | |
| | (*c*) If relevant, consider translating estimates of relative risk into absolute risk for a meaningful time period | N/A | |

Continued on next page

| | | | | |
|---|---|---|---|---|
| Other analyses | 17 | Report other analyses done—eg analyses of subgroups and interactions, and sensitivity analyses | 19 | There are no significant relationships between age and the preference for items tested in the present study, except in two cases that apply only to female participants: using an ax and a baseball bat. Older women in our sample were more likely to have the left hand at the extremity in baseball (r = .125, p = .010) and when using an ax (r = 131, p = .007). |
| **Discussion** | | | | |
| Key results | 18 | Summarise key results with reference to study objectives | 19 | In our sample, most people preferred shooting left in ice hockey. This observation is consistent with what was reported in other North American studies [4], [5],[6]. Indeed, it would seem that two thirds of right handers opt for shooting left. |
| Limitations | 19 | Discuss limitations of the study, taking into account sources of potential bias or imprecision. Discuss both direction and magnitude of any potential bias | 22-23 | There are still many avenues to explore to understand what the main factors determining lateral preference in ice hockey are and what is at stake in this hitting /control trade-off. This field of investigation would probably gain additional insights from studies dedicated to other sports that also |

require asymmetric contributions of the hands. Sports like cricket in various countries, like hurling in Ireland, or lacrosse in North America are interesting cases because of their specific requirements when holding the bat or the stick. In addition to a survey on the placement of hands in these sports by experts and novices, information could be drawn, at least for ice hockey or lacrosse, from experiments where power and control (with different tests), could be compared when hands are placed in the preferred vs. nonpreferred position.

Additional information would likely be gained from another sensory preference: a vestibular preference. There is indeed not much information about the preference for rotating in one particular direction or another along a vertical axis. Loffing et al. [1] asked their participants in which direction they would prefer rotating in figure skating and report a very poor relationship with handedness. But tasks requiring the contribution of one hand might not require as much rotation as shooting in ice hockey,

| | | | | |
|---|---|---|---|---|
| | | | | for example. It is not possible to exclude the hypothesis of a connection between a preference for rotating and the way of holding a hockey stick. What is required is to establish the link between the preference that could be observed in a test like the Fukuda's stepping test [19], for example, and the preferences for hand placements in asymmetrical tasks that also involve rotations. |
| Interpretation | 20 | Give a cautious overall interpretation of results considering objectives, limitations, multiplicity of analyses, results from similar studies, and other relevant evidence | 20-22 | In our sample, we have more people writing with their left hand than people throwing with their left hand. The writing hand is a slightly better predictor of which side one shoots in ice hockey than the hand with which one throws. However, even based on the writing hand, the choice of the grip of the hockey stick remains very variable. Indeed, while the correlation between a given unimanual task and a bimanual task is generally negative (for example, writing right would predict shooting left in hockey), there are two noticeable exceptions: when using an ax or batting in baseball. These two tasks are closely related to each other ($r_{pb}$ = .82), and negatively related to the placement of hands in ice hockey ($r_{pb}$ = -.52). The case of baseball is a documented one in the study of laterality, the game being highly lateralized [14], [15] and involving a power/control trade-off [16]. |

Contrary to the other bimanual tasks included in the study, when using an ax and a baseball bat, hands are very close to one another. This position likely favors the expression of power; there is no such power need when using a broom or a rake (some force is needed when shoveling). But the distance between hands does not explain all preferences, considering that in golf, the hands are also close to each other and the correlation with baseball ($r_{pb}$ = -.63) and using an ax ($r_{pb}$ = -.60) is negative, but positive with ice hockey ($r_{pb}$ = .71). This difference is even more noticeable that the golf club, the ax, and the baseball bat have a common characteristic: they are shorter than the tools used for other bimanual activities. Another difference between the cases of the baseball bat and ax, and the rest of bimanual activities in the study, is the fact that the latter tools require to deal with something that is on the ground (for instance, puck, leaves, snow). It is also noteworthy that for the ax, baseball, and golf, the correlations with the preferred writing hand are very low ($|r_{pb}| < .24$), but there seems to be a sex difference for the strength of the link only for using an ax or a baseball bat.

Baseball, golf, and ice hockey share the characteristic of being practiced activities. Compared to shoveling or raking leaves, sports are repeated and

| | | | | |
|---|---|---|---|---|
| | | | | practiced with the aim of improving one's performance. Through repetitions of tool manipulation (bat, club, or stick) and frequent drills, it becomes very difficult for someone to change lateral preference [17]. Some top athletes in baseball are switch-hitters, but such cases, if any, are extremely rare in ice hockey and in golf, even amongst top athletes. This implies that lateral preference in some sports is determined in the very first encounters one has with the sport, which often occurs in the early years of development. This raises the question of how a person's lateral preference is determined and crystallized. This area of the literature remains largely unexplored [18] and could be crucial for understanding lateral preference in a bimanual sport, such as ice hockey. |
| Generalisability | 21 | Discuss the generalisability (external validity) of the study results | N/A | |
| **Other information** | | | | |
| Funding | 22 | Give the source of funding and the role of the funders for the present study and, if applicable, for the original study on which the present article is based | N/A | |

*Give information separately for cases and controls in case-control studies and, if applicable, for exposed and unexposed groups in cohort and cross-sectional studies.

**Note:** An Explanation and Elaboration article discusses each checklist item and gives methodological background and published examples of transparent reporting. The STROBE checklist is best used in conjunction with this article (freely available on the Web sites of PLoS Medicine at http://www.plosmedicine.org/, Annals of Internal Medicine at http://www.annals.org/, and Epidemiology at http://www.epidem.com/). Information on the STROBE Initiative is available at www.strobe-statement.org.
